# Impact of body mass index on early and mid-term outcomes after surgery for acute Stanford type A aortic dissection

**DOI:** 10.1186/s13019-021-01558-z

**Published:** 2021-06-22

**Authors:** Yanxiang Liu, Bowen Zhang, Shenghua Liang, Yaojun Dun, Luchen Wang, Haoyu Gao, Jie Ren, Hongwei Guo, Xiaogang Sun

**Affiliations:** Department of Vascular Surgery, State Key Laboratory of Cardiovascular Disease, Fuwai Hospital, National Centre for Cardiovascular Diseases, Chinese Academy of Medical Sciences and Peking Union Medical College, No.167 North Lishi Road, Xicheng District, Beijing, 100037 China

**Keywords:** Body mass index, Acute Stanford type a aortic dissection, Frozen elephant trunk

## Abstract

**Background:**

Obesity is dramatically increasing worldwide, and more obese patients may develop aortic dissection and present for surgical repair. The study aims to analyse the impact of body mass index (BMI) on surgical outcomes in patients with acute Stanford type A aortic dissection (ATAAD).

**Methods:**

From January 2017 to June 2019, the clinical data of 268 ATAAD patients in a single centre were retrospectively reviewed. They were divided into three groups based on the BMI: normal weight (BMI 18.5 to < 25 kg/m^2^, *n* = 110), overweight (BMI 25 to < 30 kg/m^2^, *n* = 114) and obese (BMI ≥30 kg/m^2^, *n* = 44).

**Results:**

There was no statistical difference among the three groups in terms of the composite adverse events including 30-day mortality, stroke, paraplegia, renal failure, hepatic failure, reintubation or tracheotomy and low cardiac output syndrome (20.9% vs 21.9% vs 18.2% for normal, overweight and obese, respectively; *P* = 0.882). No significant difference was found in the mid-term survival among the three groups. The proportion of prolonged ventilation was highest in the obese group followed by the overweight and normal groups (59.1% vs 45.6% vs 34.5%, respectively; *P* = 0.017). Multivariable logistic regression analysis suggested that BMI was not associated with the composite adverse events, while BMI ≥30 kg/m^2^ was an independent risk factor for prolonged ventilation (OR 2.261; 95% CI 1.056–4.838; *P* = 0.036).

**Conclusions:**

BMI had no effect on the early major adverse outcomes and mid-term survival after surgery for ATAAD. Satisfactory surgical outcomes can be obtained in patients with ATAAD at all weights.

## Introduction

Obesity is causing an increase in cardiovascular morbidity, mortality, and health care cost [1, 2]. An increase in the number of obese patients appearing with acute Stanford type A aortic dissection (ATAAD) has been observed over the years. Consequently, more obese patients are likely to present for surgical repair. A few studies have explored the outcome after surgery for ATAAD in obese patients [3–5]. However, the surgical procedures in these studies were diverse and included hemi-arch replacement, total arch replacement with or without frozen elephant trunk, or even isolated ascending replacement. And the inconsistency of surgical procedures may be a confounding factor affecting the results of these studies.

At present, total arch replacement with frozen elephant trunk (TAR with FET, sometimes called Sun’s procedure) has become a routine surgical procedure to treat ATAAD in China since Sun and his colleagues introduced this surgery in 2006 [6].

The influence of obesity on surgical outcomes in ATAAD, particularly after TAR with FET, remains unclear. This study was designed to analyse the impact of varying body mass index (BMI) on early and mid-term clinical outcomes in ATAAD patients who underwent TAR with FET.

## Patients and methods

### Patients

From January 2017 to June 2019, 268 consecutive patients with ATAAD (within 14 days from the onset of symptoms to the operation) underwent TAR with FET at a single centre. All patients did not experience aortic rupture and cardiogenic shock before surgery. In our institute, TAR with FET is considered the first choice for patients with ATAAD younger than 60 years old,regardless of the location of the primary intimal tear (Hybrid surgery was performed in the patients older than 60 years old and with severe comorbidities). TAR can remove as much of the dissected aorta as possible while the FET was used to expand the true lumen, promote thrombosis of the residual false lumen and simplify the second-phase operation for the descending aorta. Patients were divided into three groups according to BMI and defined as: normal weight (BMI 18.5 to < 25 kg/m^2^), overweight (BMI 25 to < 30 kg/m^2^) and obese (BMI ≥30 kg/m^2^). Patients with BMI ≥35 kg/m^2^ were included in the obese group due to the few cases (*n* = 6). None of the patients were classified as underweight group (BMI < 18.5 kg/m^2^). This retrospective study was approved by the ethics committees of Fuwai Hospital, and written informed consent was waived.

### Surgical approach

For all patients, the previously described TAR with FET, sometimes called Sun’s procedure, was applied [7]. Sun’s procedure was performed through a standard median sternotomy under cardiopulmonary bypass (CPB) and selective cerebral perfusion (SCP) through the right axillary artery. Arterial cannulation was through the right axillary artery with the perfusion catheter directly inserted into the artery. During the cooling phase, aortic root procedures were performed if indicated. When the nasopharyngeal temperature reached 25 °C, circulatory arrest was instituted. After the three arch vessels were cross-clamped, antegrade SCP was started. To avoid recurrent laryngeal nerve injury, the aortic arch was transected between the left common carotid and left subclavian arteries. Subsequently, a stented graft was inserted into the true lumen of the descending aorta under direct vision. Then, the stented graft was anastomosed to a 4-branched graft in an end-to-end fashion using the open anastomosis technique. Distal reperfusion was initiated through the perfusion limb of the 4-branched graft once the distal anastomosis was completed. The left carotid artery was reconstructed first, followed by the ascending aorta to resume myocardial perfusion. Then the left subclavian artery was repaired, and, last, the innominate artery was repaired. After completion of the repair and adequate rewarming, the patient was weaned from CPB, and the perfusion limb of the tetrafurcated graft was ligated and divided.

### Study endpoints

The primary endpoint was composite adverse events and mid-term survival rates. Postoperative composite adverse events included 30-day mortality, stroke, paraplegia, renal failure, hepatic failure, reintubation or tracheotomy and low cardiac output syndrome. Stroke was defined as new brain injury that was clinically or radiographically evident after the procedure. Paraplegia was defined as lower limb strength less than or equal to grade 3 (able to resist gravity but not resistance). Renal failure referred to the need for haemodialysis during hospitalization. Hepatic failure was defined as a postoperative aminotransferase level exceeding 1000 IU/L. Low cardiac output syndrome referred to the need for an intra-aortic balloon pump.

The secondary endpoint was prolonged ventilation which was defined as a postoperative intubation time more than 24 h.

### Statistical analysis

Data were reported as the mean ± standard deviation or median with an interquartile range (IQR) for quantitative variables and as frequencies and percentages for categorical variables. For quantitative data, one-way ANOVA was used for normally distributed values and Kruskal-Wallis H test was applied for abnormally distributed values with or without homogeneity of variance. For categorical data, the Pearson χ^2^ test or Fisher’s exact test was used to evaluate the differences in percentage. Survival was analysed by using the Kaplan-Meier method and log-rank tests.

Multivariable logistic regression analysis was applied to identify the risk factors for composite adverse events and prolonged ventilation. All potential covariates of interest were included in a univariable logistic regression model. The multivariable logistic regression model included significant variables (*P* < 0.1) in the univariable logistic regression. All the statistical tests were 2-sided, and a *P* value of < 0.05 was considered to indicate statistical significance. All statistical analyses were done using SPSS version 25 (IBM, Armonk, NY).

## Results

### Patient characteristics

Patient characteristics are summarized in Table 1. Of the 268 patients, 110 (41.0%) patients were classified as normal weight, 114 (42.5%) patients as overweight, 44 (16.4%) patients as obese. Compared to normal weight patients, overweight and obese patients had a larger proportion of male gender, though the difference was not significant (*P* = 0.070). There was no significant difference among the three groups in terms of age, hypertension, coronary artery disease, diabetes, cerebrovascular events, chronic kidney disease, New York Heart Association class ≥3, malperfusion syndrome, left ventricular ejection fraction and median or massive aortic regurgitation (*P* > 0.05).

**Table 1:** Baseline patient characteristics

Variables	Normal (*n* = 110)	Overweight (*n* = 114)	Obese (*n* = 44)	*P* value
Age (years)	47.1 ± 9.4	45.2 ± 9.5	45.2 ± 7.4	0.257
Male	82 (74.5)	92 (80.7)	40 (90.9)	0.070
Hypertension	89 (80.9)	94 (82.5)	39 (88.6)	0.519
CAD	16 (14.5)	21 (18.4)	12 (27.3)	0.182
Diabetes	2 (1.8)	4 (3.5)	2 (4.5)	0.578
Cerebrovascular events	7 (6.4)	6 (5.3)	1 (2.3)	0.588
CKD	2 (1.8)	0 (0)	1 (2.3)	0.298
NYHA≥3	4 (3.6)	5 (4.4)	1 (2.3)	1.000
Organ malperfusion				
Cardiac	5 (4.5)	7 (6.1)	3 (6.8)	0.824
Cerebral	5 (4.5)	6 (5.3)	1 (2.3)	0.859
Visceral	2 (1.8)	1 (0.9)	1 (2.3)	0.668
Limb	3 (2.7)	4 (3.5)	2 (4.5)	0.820
LVEF	60 (4)	60 (3)	60 (3)	0.856
Moderate-to-severe AR	30 (27.3)	30 (26.3)	10 (22.7)	0.851

*CAD* coronary artery disease, *CKD* chronic kidney disease, *NYHA* New York Heart Association, *LVEF* left ventricular ejection fraction, *AR* aortic regurgitation

Hypertension is defined as a systolic blood pressure greater than 140 mmHg

### Operative data

Operative details are listed in Table 2. TAR with FET was performed in all the patients. No significant difference was found in the concomitant surgeries. Also, cardiopulmonary bypass time, cross-clamp time and circulatory arrest time were close among the three groups. No significant difference was found among the three groups in the transfusion amount including red blood cells, fresh frozen plasma and platelets.

**Table 2:** Operative details

Variables	Normal (*n* = 110)	Overweight (*n* = 114)	Obese (*n* = 44)	*P* value
Combined surgery				
Bentall	32 (29.1)	30 (26.3)	9 (20.5)	0.547
Sinus reconstruction	33 (30.0)	45 (39.5)	21 (47.7)	0.091
CABG	18 (16.4)	21 (18.4)	12 (27.3)	0.290
Wheat’s	5 (4.5)	1 (0.9)	0 (0)	0.145
Other (David, Mitral, Congenital)	7 (6.4)	2 (1.8)	2 (4.5)	0.186
CPB time (min)	166.0 (59.0)	174.0 (78.0)	172.0 (71.0)	0.511
Cross-clamp time (min)	109.0 (48.0)	112.0 (50.0)	101.5 (52.0)	0.656
Circulatory arrest time (min)	16.0 (3.0)	16.0 (4.0)	16.5 (3.0)	0.517
Transfusion				
Red blood cells (U)	0 (0)	0 (0)	0 (0)	0.350
Fresh frozen plasma (ml)	400 (600)	400 (600)	400 (600)	0.999
Platelets (U)	1 (1)	1 (0)	1 (0)	0.105

Bentall procedure was performed in the patients with severely dilated roots, or with the roots severely involved by the dissection

### Outcome characteristics

Table 3 summarizes the early postoperative outcomes. The rates of composite adverse events were comparable among groups (20.9% vs 21.9% vs 18.2% for normal, overweight and obese, respectively; *P* = 0.882). And there were no significant differences among the three groups in terms of 30-day mortality, stroke, paraplegia, renal failure, hepatic failure, reintubation or tracheotomy or low cardiac output syndrome.

**Table 3:** Early postoperative outcomes

Variables	Normal (*n* = 110)	Overweight (*n* = 114)	Obese (*n* = 44)	*P* value
Composite adverse events	23 (20.9)	25 (21.9)	8 (18.2)	0.882
30-day mortality	9 (8.2)	4 (3.5)	2 (4.5)	0.305
Stroke	3 (2.7)	4 (3.5)	2 (4.5)	0.820
Paraplegia	2 (1.8)	8 (7.0)	1 (2.3)	0.132
Renal failure	12 (10.9)	13 (11.4)	5 (11.4)	1.000
Hepatic failure	8 (7.3)	8 (7.0)	3 (6.8)	1.000
Reintubation or tracheotomy	2 (1.8)	4 (3.5)	1 (2.3)	0.875
Low cardiac output syndrome	3 (2.7)	1 (0.9)	1 (2.3)	0.487
ICU time (h)	89.6 (66.8)	90.1 (76.3)	90.4 (91.8)	0.484
In-hospital time (d)	10.0 (5.0)	12.0 (6.0)	11.0 (7.0)	0.173
Ventilation time (h)	20.9 (22.9)	22.0 (44.8)	30.4 (60.7)	0.039
Ventilation time>24 h	38 (34.5)	52 (45.6)	26 (59.1)	0.017

The ICU time and the in-hospital time did not differ among groups. The ventilation time in the obese group was significantly longer than that in the normal weight and overweight group (20.9 h vs 22.0 h vs 30.4 h for normal, overweight and obese, respectively; *P* = 0.039). What’s more, the proportion of ventilation time > 24 h in the overweight and obese patients was significantly higher than that in the normal weight patients, and the higher the obesity, the higher the proportion of ventilation duration > 24 h (34.5% vs 45.6% vs 59.1% for normal, overweight and obese, respectively; *P* = 0.017).

**Fig. 1:**
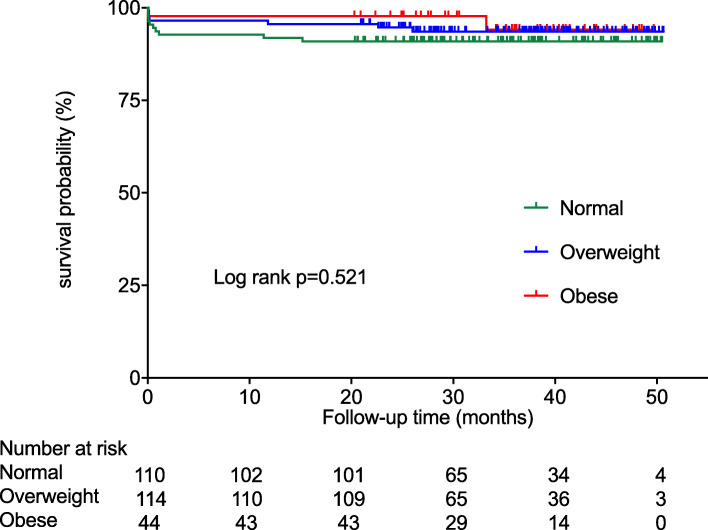
Kaplan-Meier analysis of the overall survival in normal weight (BMI 18.5 to < 25 kg/m^2^), overweight (BMI 25 to < 30 kg/m^2^) and obese (BMI ≥30 kg/m^2^) patients with acute type A aortic dissection. BMI, body mass index

Finally, no statistical difference was found among the BMI groups regarding the mid-term survival (Fig. 1; log-rank test, *P* = 0.521).

### Regression analysis

The regression analysis for composite adverse events is showed in Table 4. According to the univariable and multivariable analysis, BMI ≥25 kg/m^2^ or BMI ≥30 kg/m^2^ were not associated with composite adverse events. The independent predictors of composite adverse events were cardiac malpersuion (OR 4.019; 95% CI 1.128–14.322; *P* = 0.032), cerebral malpersuion (OR 5.060; 95% CI 1.402–18.268; *P* = 0.013), and limb malpersuion (OR 10.223; 95% CI 2.266–46.124; *P* = 0.002).

**Table 4:** Univariable and multivariable logistic analysis for risk factors associated with composite adverse events

Risk factor	Univariable analysis		Multivariable analysis	
	OR (95% CI)	*P* value	OR (95% CI)	*P* value
Age (years)	1.009 (0.977–1.042)	0.578		
Male	1.104 (0.536–2.273)	0.788		
BMI (kg/m^2^)				
BMI < 25	1.000			
BMI ≥ 25	1.063 (0.561–2.013)	0.852		
BMI ≥ 30	0.841 (0.344–2.054)	0.703		
Hypertension	0.700 (0.335–1.462)	0.343		
CAD	3.115 (1.587–6.116)	0.001	1.440 (0.301–6.895)	0.648
Diabetes	1.272 (0.250–6.478)	0.772		
Cerebrovascular events	1.034 (0.279–3.841)	0.960		
CKD	NS	NS		
NYHA≥3	1.658 (0.415–6.628)	0.475		
Organ malperfusion				
Cardiac	4.881 (1.688–14.116)	0.003	4.019 (1.128–14.322)	0.032
Cerebral	4.120 (1.275–13.315)	0.018	5.060 (1.402–18.268)	0.013
Visceral	1.267 (0.129–12.416)	0.839		
Limb	8.360 (2.021–34.581)	0.003	10.223 (2.266–46.124)	0.002
LVEF	0.970 (0.903–1.042)	0.411		
Median or massive AR	0.551 (0.261–1.162)	0.117		
Bentall	1.142 (0.592–2.201)	0.692		
CABG	3.640 (1.873–7.076)	< 0.001	1.675 (0.331–8.460)	0.533
CPB time	1.010 (1.005–1.014)	< 0.001	1.004 (0.999–1.010)	0.116
Cross-clamp time	1.006 (0.999–1.013)	0.121		
Circulatory arrest time	1.045 (0.963–1.134)	0.294		
Transfusion				
Red blood cells	1.274 (1.122–1.447)	< 0.001	1.144 (0.970–1.348)	0.109
Fresh frozen plasma	1.001 (1.000–1.002)	0.002	1.000 (1.000–1.001)	0.308
Platelets	1.196 (0.947–1.511)	0.134		

*OR* odds ratio, *CI* confidence interval, *NS* no significance

In the univariable regression analysis for ventilation time > 24 h (Table 5), several variables were associated with ventilation time > 24 h (*P* < 0.1). BMI ≥30 kg/m^2^ (OR 2.261; 95% CI 1.056–4.838; *P* = 0.036), moderate-to-severe aortic regurgitation (OR 0.492; 95% CI 0.249–0.971; *P* = 0.041) and transfusion amount of fresh frozen plasma (OR 1.001; 95% 1.000–1.001; *P* = 0.019) were identified as risk factors in the multivariable regression analysis.

**Table 5:** Univariable and multivariable logistic analysis for risk factors associated with ventilation time>24 h

Risk factor	Univariable analysis		Multivariable analysis	
	OR (95% CI)	*P* value	OR (95% CI)	*P* value
Age (years)	1.011 (0.985–1.039)	0.398		
Male	0.878 (0.479–1.609)	0.673		
BMI (kg/m^2^)				
BMI < 25	1.000		1.000	
BMI ≥ 25	1.589 (0.927–2.724)	0.092	1.565 (0.890–2.751)	0.120
BMI ≥ 30	2.737 (1.335–5.612)	0.006	2.261 (1.056–4.838)	0.036
Hypertension	1.474 (0.758–2.865)	0.253		
CAD	2.442 (1.293–4.610)	0.006	1.508 (0.353–6.440)	0.579
Diabetes	0.427 (0.085–2.155)	0.303		
Cerebrovascular events	1.802 (0.608–5.347)	0.288		
CKD	0.652 (0.058–7.281)	0.728		
NYHA≥3	0.869 (0.239–3.154)	0.831		
Organ malperfusion				
Cardiac	0.867 (0.300–2.508)	0.792		
Cerebral	0.422 (0.112–1.595)	0.203		
Visceral	0.432 (0.044–4.206)	0.470		
Limb	2.709 (0.663–11.070)	0.165		
LVEF	1.021 (0.959–1.087)	0.510		
Median or massive AR	0.465 (0.260–0.832)	0.010	0.492 (0.249–0.971)	0.041
Bentall	0.536 (0.303–0.948)	0.032	0.746 (0.383–1.453)	0.389
CABG	2.176 (1.170–4.047)	0.014	1.549 (0.374–6.421)	0.546
CPB time	1.002 (0.999–1.006)	0.249		
Cross-clamp time	0.999 (0.993–1.005)	0.696		
Circulatory arrest time	1.051 (0.978–1.129)	0.174		
Transfusion				
Red blood cells	1.094 (0.979–1.223)	0.113		
Fresh frozen plasma	1.001 (1.000–1.001)	0.008	1.001 (1.000–1.001)	0.019
Platelets	1.305 (0.987–1.726)	0.062	1.313 (0.948–1.818)	0.101

*OR* odds ratio, *CI* confidence interval

## Discussion

The main findings of the study were that obese patients with ATAAD undergoing TAR with FET achieve comparable early outcomes compared to normal weight and overweight patients; obese patients require longer ventilation time than normal weight and overweight patients.

Obesity has long been recognised as a major risk factor for cardiovascular diseases including hypertension, coronary artery disease and heart failure [8, 9]. However, the impact of obesity on cardiac surgery remains controversial. Some authors reported that obesity was related to worse outcomes after open heart surgery, with higher early mortality, worse late survival and increased rate of postoperative complications including renal failure, prolonged intensive care unit stay and sternal wound infection [1, 10–13]. While others have suggested the existence of a protective ‘obesity paradox’ in cardiac surgery, defined as a protective effect of obesity against early postoperative complications and late mortality [2, 14–17]. The influence of obesity on aortic dissection surgery appears to be similar in the current study. Maximilian Kreibich and his colleagues reported that obesity was not associated with a greater risk of death or other adverse outcomes in patients undergoing surgery for type A aortic dissection [3]. Yang Li and his colleagues also reported that BMI had no effect on in-hospital death and postoperative complications after open surgery for acute thoracic aortic dissection [4]. Consistent with these findings, the composite adverse events were also not associated with obesity in our study. In our previous article designed to explore the risk factors for major adverse outcomes in ATAAD, BMI was not identified as a risk factor. And the major adverse outcomes were more related to age, malperfusion syndrome and CPB time, which was in line with our current study [18].

Obesity represents a significant problem for the respiratory system, causing a number of physiological changes. Obesity is related to impaired pulmonary function, including increased residual lung volume, decreased lung compliance and increased chest wall impedance, ventilation-perfusion abnormalities, depressed ventilatory drive, and bronchospasm [19]. During postoperative ventilation, atelectasis is a significant problem for obese patients, which is associated to paralysis, sedation, and supine positioning [20, 21]. Moreover, obesity predisposes to obstructive sleep apnoea syndrome, which is a risk factor for difficult ventilation [22–24]. Therefore, obese patients are at increased risk of postoperative respiratory complications and require more specific respiratory management after surgery, which is reflected in our study as prolonged ventilation time in overweight and obese patients. This phenomenon has also been observed by Maximilian Kreibich and Yang Li in their studies [3, 4]. In addition, we found that moderate-to-severe aortic regurgitation and transfusion volume of fresh frozen plasma are the risk factors for prolonged ventilation time. This suggests that pulmonary congestion and pulmonary edema caused by aortic regurgitation and intraoperative fluid infusion are the reasons for decreased respiratory function.

Maximilian Kreibich and his colleagues reported similar long-term survival among all BMI groups in patients with type A aortic dissection, which was consistent with our findings [3]. Although obesity may increase the risk of hypertension, coronary heart disease, diabetes, etc., the long-term survival of patients with aortic dissection is mainly related to the progression of residual dissection, which is profoundly influenced by the location and size of remained tears [25]. This may explain the similar mid-term survival among the BMI groups.

### Limitations

There are some limitations in our study. This study is limited by its non-randomized, single-centre, retrospective nature. Furthermore, the sample size was small, especially for obese group. There were no underweight and few morbidly obese (BMI ≥35 kg/m^2^) patients in this study, which may be due to the demographic characteristics of China. Lastly, the long-term prognosis of patients with ATAAD in different groups should also be of concern. This study only reported the early and mid-term outcomes. Future studies should pay more attention to late complications, quality of life, and cause of death.

## Conclusions

BMI had no effect on the major adverse outcomes and mid-term survival after surgery for ATAAD. Satisfactory surgical outcomes can be obtained in patients with ATAAD at all weights.

## Data Availability

The datasets used and/or analyzed during the current study are available from the corresponding author on reasonable request.

## Abbreviations

BMI: body mass index
ATAAD: acute Stanford type A aortic dissection
TAR with FET: total arch replacement with frozen elephant trunk
CPB: cardiopulmonary bypass
SCP: selective cerebral perfusion
CABG: coronary artery bypass grafting
